# Disseminated abscesses from *Treponema denticola* diagnosed by next-generation sequencing: case report

**DOI:** 10.1515/biol-2025-1387

**Published:** 2026-10-12

**Authors:** Xuhong Zhang, Shanshan Yu, Ye Liu

**Affiliations:** Department of Nursing, The First Affiliated Hospital, Zhejiang University School of Medicine, Hangzhou 310000, China

**Keywords:** *Treponema denticola*, disseminated abscesses, next-generation sequencing, odontogenic infections

## Abstract

*Treponema denticola* is a fastidious oral spirochete that is difficult to detect by conventional culture, and extraoral disseminated infections are exceedingly rare. We report a rare case of disseminated abscesses involving the liver, kidney and pararenal regions caused by *T. denticola*, highlighting the role of next-generation sequencing (NGS) in diagnosis of uncommon abscesses. A 50-year-old female presented with a refractory lumbar abscess. Initial conventional cultures were negative, and empirical antibiotic therapy was ineffective. Abdominal computed tomography (CT) revealed liver abscess, and multiple renal and hepatic abscess. After the initial NGS analysis of the abscess fluid led to an inappropriate treatment adjustment, repeat NGS analysis of a liver biopsy specimen identified *T. denticola.* The patient’s condition was alleviated after the administration of a combined penicillin and doxycycline regimen and radical resection of the right hepatic lobe and kidney. This case confirms that *T. denticola* can cause severe systemic infection. The integration of advanced diagnostic technologies and multidisciplinary collaboration is crucial in managing infections of unknown origin.

## 1 Introduction

*Treponema denticola* is an anaerobic, gram-negative oral spirochete implicated in the pathogenesis and progression of periodontal diseases [1]. In addition to its role in oral health, *T. denticola* has also been associated with the development of several systemic diseases, including atherosclerosis, pneumonia, and even neurodegenerative disorders, particularly when oral bacteria disseminate through the bloodstream [2], [3], [4]. However, the underlying mechanisms remain poorly understood because of the complex interplay of multiple factors, including the secretion of tissue-degrading proteases, activation of inflammatory responses, and evasion of host immune defenses [3], 5].

Although *T. denticola* has been implicated in several diseases, its diagnosis remains challenging, particularly deep-seated abscesses as these infections often lack distinctive clinical manifestations and the organism is difficult to identify using conventional culture-based methods. Next-generation sequencing (NGS) has substantially improved the detection of uncommon and fastidious pathogens in diagnostically challenging infections. In particular, NGS has facilitated the identification of *T. denticola* in complex infections that might otherwise remain undiagnosed [6]. Previous studies have reported the detection of *T. denticola* in pulmonary infections, expanding the recognized pathogenic spectrum of this organism beyond the oral cavity [2], 7]. These advances in molecular diagnosis have increased awareness of extraoral infections caused by *T. denticola*; nevertheless, disseminated infections involving multiple organs remain exceptionally rare.

Herein, we report a rare case of disseminated abscesses involving the liver, kidney and pararenal region caused by *T. denticola*, with the pathogen identified by NGS. This case expands the clinical spectrum of *T. denticola* infection and highlights its potential to cause disseminated deep-seated infections.

## 2 Case presentation

A 50-year-old lady presented to our hospital in December 2022 with a refractory lumbar abscess. The condition had initially developed one month earlier, and she had undergone surgical drainage at a local hospital; however, the response was unsatisfactory.

Physical examination revealed a healed scar in the lumbar region without exudate. A palpable mass measuring approximately 2 × 3 cm was detected adjacent to the scar. The mass was characterized by limited mobility, mild tenderness on palpation, and the absence of fluctuation. Abdominal computed tomography (CT) demonstrated a hepatic abscess, infectious lesions associated with calculi and hydronephrosis in the right kidney, as well as sinus tract in the pararenal region secondary to infection (Figure 1A–D).

**Figure 1:**
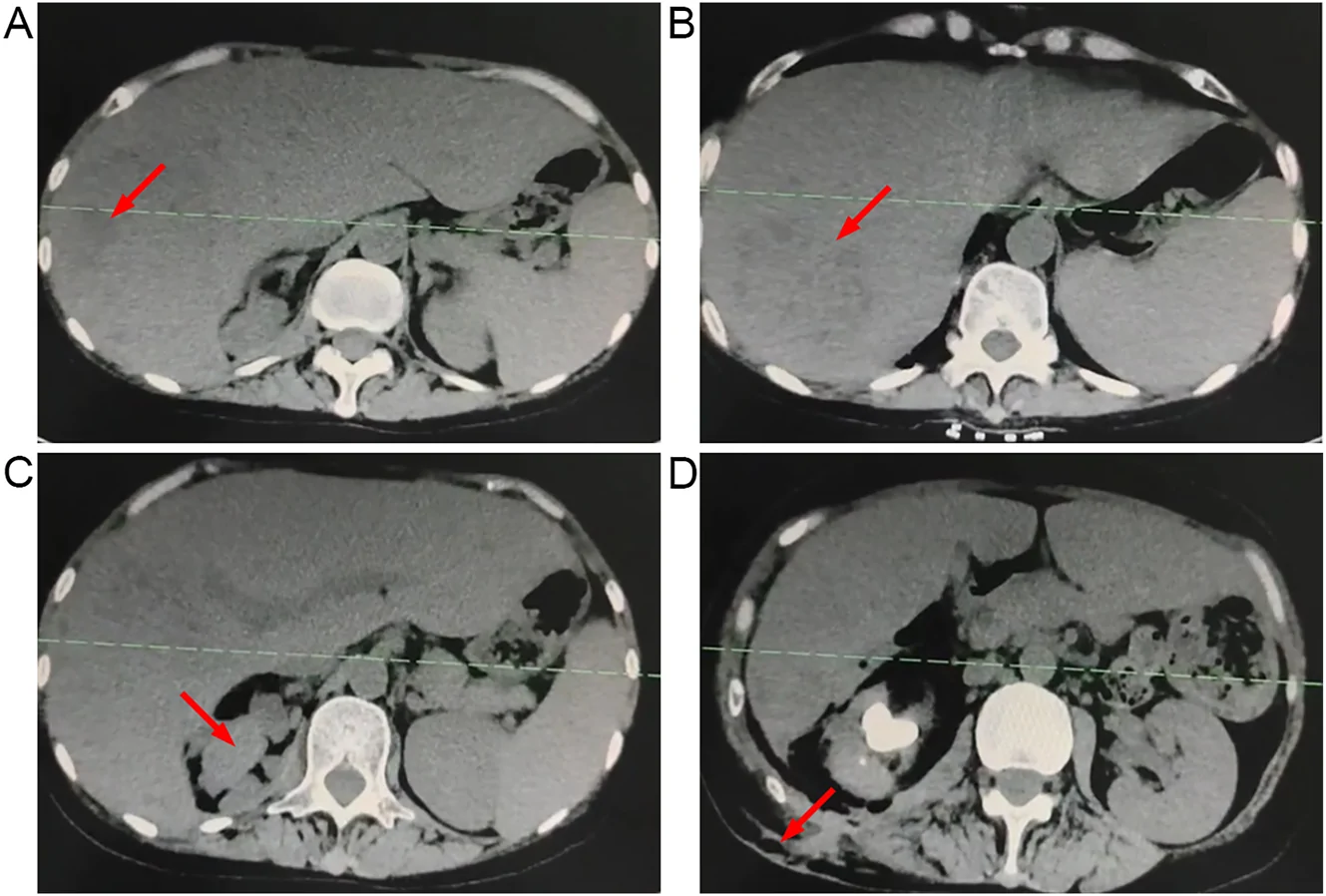
Abdominal CT scan revealed liver abscess and an infectious lesion in the right kidney. (A, B) A space-occupying lesion in the right lobe of the liver. (C) A patchy shadow is noted at the upper border of the right kidney, with an infective lesion being suspected.​​ (D) A patchy shadow is noted in the right lumbar region, suggestive of an inflammatory lesion with associated sinus tract formation.​​

Routine microbiological examinations were performed on blood, urine, and wound exudate specimens. Routine cultures of urine, blood, and wound exudate were negative. Therefore, empirical antibacterial therapy with piperacillin–tazobactam (4.5 g every 8 h) was initiated. However, the wound lesions showed no meaningful improvement. Subsequently, the blood T-SPOT.TB test returned positive, while repeated aerobic and anaerobic cultures and wound-smear examinations remained negative. Tuberculosis was therefore considered a possible diagnosis at that stage, and piperacillin–tazobactam was discontinued. The treatment regimen was changed to standard four-drug antituberculosis therapy consisting of isoniazid (300 mg/day), rifampicin (450 mg/day), pyrazinamide (1,500 mg/day) and ethambutol (750 mg/day), together with cefuroxime (1,500 mg, twice a day) and linezolid (600 mg, twice a day). Despite this treatment, the patient’s condition continued to deteriorate, with persistent exudation from the lumbar and back wounds, progressive enlargement of the sinus tract, and increased discharge from the lesion.

In December 2022, the patient was transferred to our department because of multiple abscesses. On physical examination, the lumbar sinus opening had enlarged and was associated with purulent discharge. Vital signs and physical examination findings were similar to those at the previous admission, with no significant abnormalities detected on cardiovascular, respiratory, or abdominal examinations. Laboratory investigations revealed a white blood cell count of 12.22 × 10^9^/L (neutrophils, 81 %; lymphocytes, 7.9 %), a C-reactive protein of 143 mg/L, and a hemoglobin level of 63 g/L. Chest CT demonstrated right lower lobe pneumonia with a small pleural effusion. Contrast-enhanced abdominal CT revealed a hepatic abscess measuring 11.5 × 7.2 cm, multiple abscesses in the right kidney, and a sinus tract in the lumbar region (Figure 2A–D).

**Figure 2:**
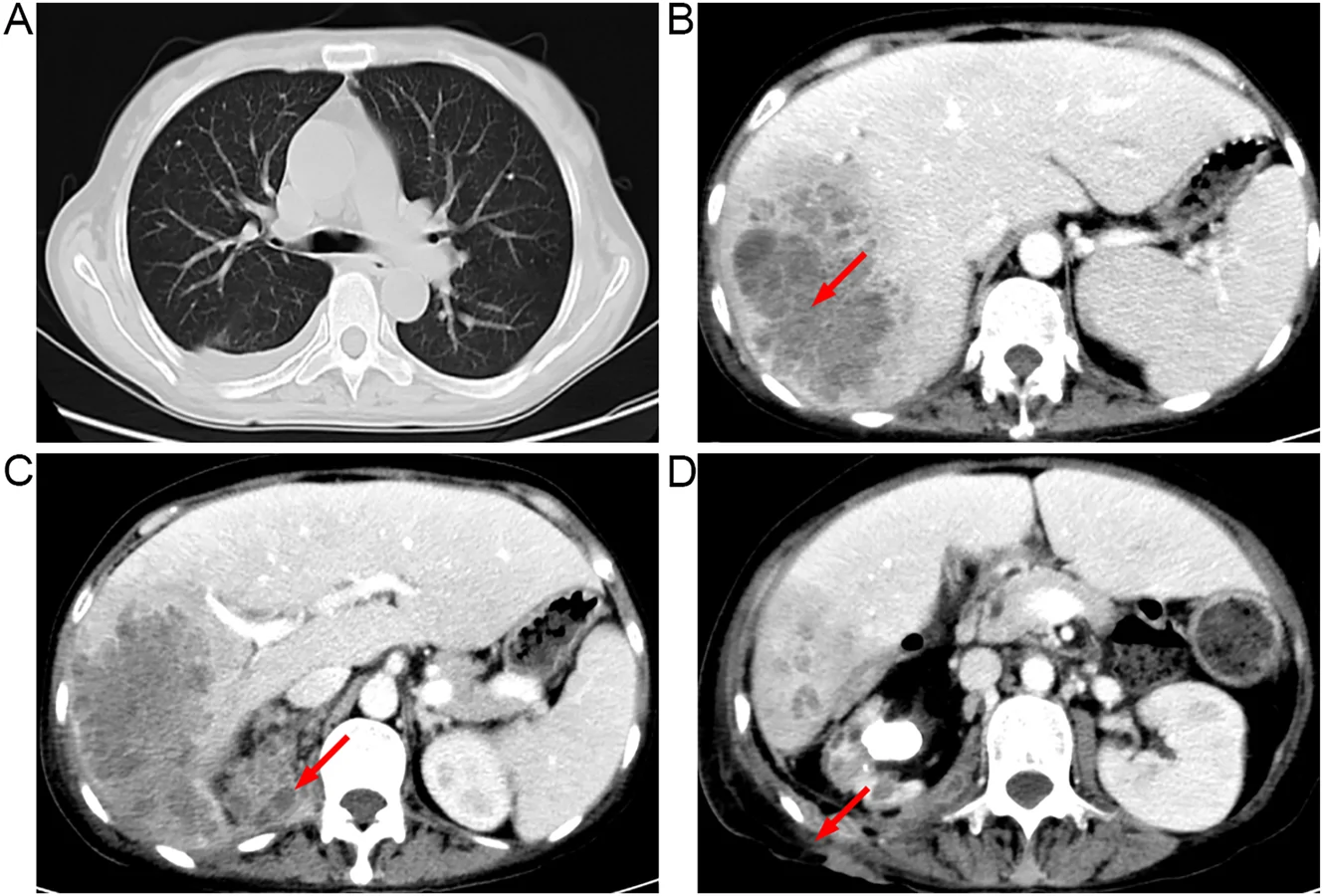
Chest CT and contrast-enhanced abdominal CT after admission. (A) Inflammation in the right lower lobe of the lung with pleural effusion. (B) Liver abscess in the right lobe. (C, D) multiple abscesses formed in the right kidney and its upper region, the right perirenal posterior space, the right diaphragmatic crus, and the psoas major muscle, with sinus tract formation in the right lumbar back.

Five days after the initiation of antituberculosis therapy, ultrasound-guided percutaneous catheter drainage of the hepatic abscess and aspiration of the perinephric abscess were performed. Peripheral blood and urine samples were examined using conventional microbiological methods, while abscess fluid obtained from the lumbar and hepatic lesions was submitted for both conventional culture and NGS. Total genomic DNA was extracted from each specimen using the DNeasy Blood & Tissue Kit (Qiagen, Hilden, Germany) according to the manufacturer’s instructions. DNA libraries were prepared using the Illumina DNA Prep Kit following the manufacturer’s protocol. Sequencing was performed on an Illumina NovaSeq 6,000 platform using paired-end 150 bp (PE150) chemistry. After quality control, host-derived sequences were removed by aligning reads to the human reference genome (GRCh38) using Bowtie2 (v2.5.0). A no-template control (NTC) and extraction blank were processed in parallel throughout DNA extraction, library preparation, and sequencing. Microorganisms detected in the negative controls were regarded as potential contaminants and excluded from downstream analyses. Bacterial and *M. tuberculosis* cultures remain negative. NGS detected several microorganisms in the abscess fluid, among which *Propionibacterium* spp. had the highest number of sequence reads (187 reads) and was the predominant organism identified. Based on the NGS findings, previous treatment regimen was adjusted to moxifloxacin, meropenem, linezolid, and sulfamethoxazole. Despite drainage and the modified antimicrobial regimen, the patient developed persistent fever.

Liver biopsy revealed partial hepatic tissue with cloudy swelling degeneration and stromal fibrous proliferation, accompanied by focal areas of complete necrosis (Figure 3A and B). Immunohistochemical analysis showed positive staining for pan-cytokeratin in epithelial cells and hepatocytes, suggesting a possible stem cell origin of the lesion. Common hepatocellular carcinoma and infectious etiologies were excluded, as glypican-3, fungal immunofluorescence, tuberculosis fluorescence, and acid-fast staining were all negative (data not shown).

**Figure 3:**
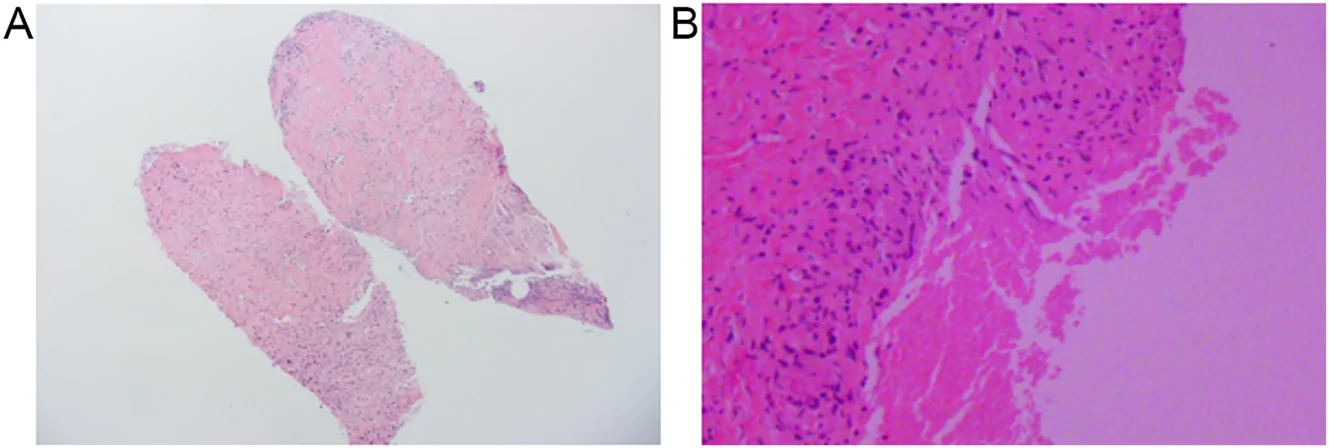
HE staining results showed proliferation of interstitial fibrous tissue and focal coagulative necrosis. (A) The hepatocytes are well differentiated, with noticeable proliferation of stromal fibrous tissue. (B) Focal complete necrosis is present.

NGS analysis of the liver biopsy specimen identified *T. denticola*, with 33 species-specific sequence reads detected, suggesting its potential clinical relevance in the infection. Subsequent oral examination revealed concomitant periodontitis. The antimicrobial regimen was therefore adjusted to include penicillin (1.6 × 10^6^U, three times a day) and doxycycline (100 mg, twice a day) for targeted treatment of *T. denticola*​ infection. Following initiation of targeted antimicrobial therapy, the patient’s inflammatory markers decreased, and the body temperature returned to normal. However, follow-up CT demonstrated persistent large hepatic and renal abscesses, necessitating surgical intervention.

Following multidisciplinary team (MDT) consultations involving hepatobiliary surgery, urology, radiology, ultrasound interventional therapy, and infectious diseases, surgical treatment was recommended, including right hepatic lobectomy and right nephrectomy. The surgery was successfully performed, and follow-up examinations after treatment showed normalization of inflammatory markers and no abnormalities on follow-up CT scans (Figure 4A and B).

**Figure 4:**
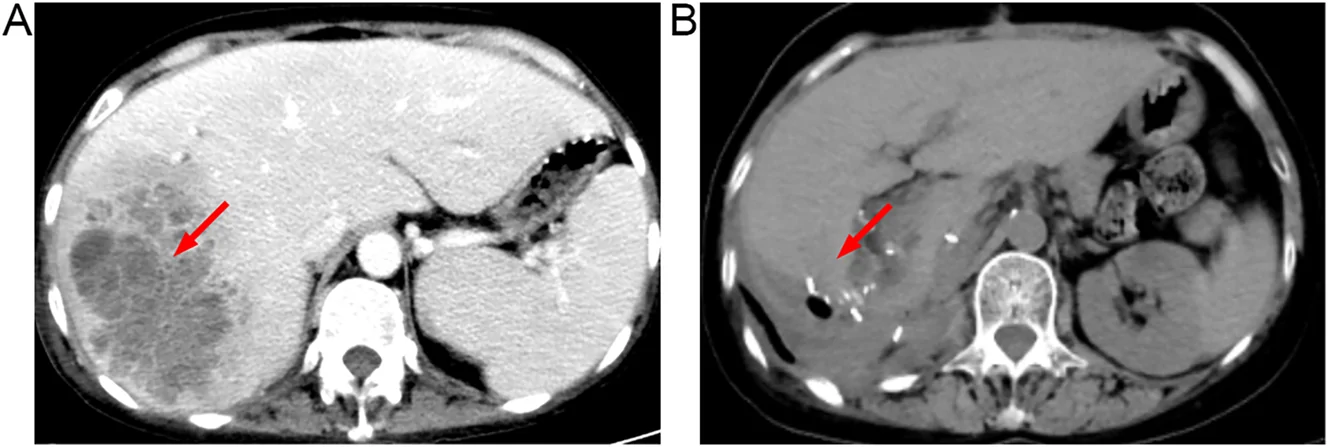
The follow-up CT results reveal that liver abscess is no longer visible. (A) Preoperative abdominal CT scan. ​(B) follow-up abdominal CT scan.

**Ethical approval:** The research related to human use has been complied with all the relevant national regulations, institutional policies and in accordance with the tenets of the Helsinki Declaration, and has been approved by the Ethics Committee of the First Affiliated Hospital, Zhejiang University School of Medicine (Approval No.: 1251).

**Informed consent:** Informed consent was obtained from all individuals participating in this study, or from their legal guardians or wards.

## 3 Discussion

*T. denticola* can disseminate beyond the oral cavity and has been implicated in the pathogenesis of several systemic diseases, including pneumonia, neurodegenerative disorders, and atherosclerosis. To our knowledge, based on the available literature, this is the first reported case of disseminated *T. denticola* infection presenting with multiple abscesses involving the lungs, liver, kidneys, and skeletal muscles. The diagnosis was established by NGS, underscoring the importance of molecular diagnostic techniques in identifying rare anaerobic pathogens and facilitating timely targeted antimicrobial therapy.

As a member of the red complex bacteria, *T. denticola* can interact synergistically with *Porphyromonas gingivalis* and *Tannerella forsythia*, contributing to the development and progression of periodontitis, pulpitis, and periapical diseases [8], 9]. Oral bacteria are not only implicated in local oral diseases but have also been associated with systemic infections, cardiovascular diseases, gastrointestinal inflammation, adverse pregnancy outcomes, and neurodegenerative disorders [10], [11], [12], [13], [14], [15]. These pathogenic microorganisms may disseminate through the bloodstream or lymphatic system, potentially leading to secondary infections at distant sites. Therefore, dental infections should be considered a potential source of infection in patients presenting with unexplained systemic infections. Although abscesses associated with *T. denticola* are uncommon, a few cases have been reported. In 2009, a case of liver abscess was reported in which several periodontal pathogens, including *T. denticola*, were identified [16]. In another case, a 60-year-old male presented with a periorbital mass, and biopsy revealed inflammatory changes and spiral-shaped bacteria resembling *Treponema* species [17]. In this case, we reported a rare case of multiple abscesses involving the lungs, liver, kidneys, and muscular tissue, in which *T. denticola* was identified by NGS.

Due to the lack of distinctive imaging and clinical manifestations, the diagnosis of multiple abscesses caused by *T. denticola* depends on bacterial culture results. However, repeated cultures of blood, urine, and abscess fluid samples failed to identify any pathogens in this case. In fact, the oral cavity harbors numerous anaerobic bacteria similar to *T. denticola*, which require specialized culture conditions and grow slowly, thereby hampering timely identification of the pathogen [18]. Traditional pathogen detection methods, such as morphological observation culture-based isolation, biochemical testing, and immunological assays, have limited sensitivity and specificity, especially for rare microorganisms. Clinically, bacterial culture and molecular methods like PCR are commonly used to detect pathogens such as *T. denticola* in oral infections, but this strategy is not suitable for secondary infections [19]. Recently, NGS enables high-throughput sequencing of nucleic acids directly extracted from clinical specimens such as blood, urine, or abscess fluid, allowing rapid and unbiased detection of diverse pathogens through sequence comparison with reference databases [20]. Nevertheless, NGS results should always be interpreted in conjunction with the patient’s clinical presentation, imaging findings, and other microbiological evidence to distinguish true pathogens from colonizing or contaminating microorganisms. This technology significantly shortens the diagnostic time and enhances the accuracy and efficiency of clinical diagnosis. Numerous clinical reports have confirmed the utility and reliability of NGS in diagnosing odontogenic infections. Some cases, including the present one, involved patients without obvious oral symptoms or periodontal surgery history, yet NGS still detected oral microorganisms in samples [21]. This phenomenon may result from empirical antibiotic treatment administered in the early stages, which partially alleviated periodontal symptoms but set barriers for clinical confirmation of odontogenic origin. Notably, in this case, the correct pathogen was identified after two rounds of NGS testing, with results differing from the initial conventional pathogen assays. Although previous studies have demonstrated that NGS performed on abscess fluid exhibits high sensitivity and accuracy [22], 23], the causative pathogen in our patient was ultimately identified by NGS performed on tissue specimens. The discrepancy between the two NGS results may reflect several factors. First, the specimens were obtained from different sites, and microbial composition may vary according to the sampling location. Second, differences in bacterial burden may have affected pathogen detection. In addition, prior antimicrobial therapy could have altered the relative abundance of microorganisms and influenced sequencing results. Therefore, the initial detection of *Propionibacterium* may have represented a low-abundance or non-dominant organism, whereas the subsequent identification of *T. denticola* in the liver biopsy specimen, together with the clinical response to targeted therapy, supported its greater clinical relevance as the causative pathogen.

The accurate diagnosis of the pathogen and the successful implementation of subsequent treatment are also attributed to MDT consultations. In this case, all traditional detections yielded negative results, and empirical treatments showed ineffective outcomes. Following multidisciplinary discussion, NGS was selected as the next diagnostic approach to identify the causative pathogen. Once the pathogen was identified, the MDT provided a comprehensive treatment strategy capable of resolving all abscesses in a single intervention and adjusted the medication immediately, which is highly effective. The successful treatment highlights the importance of MDT meetings, especially in cases of refractory multiple infections with unidentified pathogens.

This case has inherent limitations. Data derived from a single patient cannot be generalized to represent broader populations. Larger-scale studies are needed to validate these findings. We acknowledge that clinical improvement after antimicrobial therapy cannot completely exclude the presence or contribution of other anaerobic microorganisms, particularly when the administered regimen also has activity against other oral anaerobes. Finally, the complete species-level abundance profile and raw data required to generate a reliable Krona chart or stacked bar plot were not available. Therefore, we were unable to reconstruct the overall metagenomic composition retrospectively.

## 4 Conclusions

This case of disseminated abscesses caused by *T. denticola* highlights the potential of oral anaerobes to cause systemic infections. NGS analysis of specimens from multiple infection sites can substantially improve the speed and accuracy of diagnosing rare and complex infections. MDT collaboration was crucial in designing an individualized diagnostic and therapeutic plan. This combined strategy can improve diagnostic efficiency and optimize treatment outcomes for complex infectious cases.

## References

[1] Goetting-Minesky MP, Godovikova V, Saraithong P, Rickard AH, Crawley BR, Agolli SM (2026). Expanded functional characterization and optimization of protein expression in Treponema denticola shuttle plasmids. Mol Oral Microbiol.

[2] Guo Y, Zhu Y, Jiang L (2025). Treponema denticola as a potential pathogen of pneumonia in individuals with poor oral hygiene: a case report. Eur J Clin Microbiol Infect Dis.

[3] Pisani F, Pisani V, Arcangeli F, Harding A, Singhrao SK (2023). Treponema denticola has the potential to cause neurodegeneration in the midbrain via the periodontal route of infection-narrative review. Int J Environ Res Publ Health.

[4] Ciccotosto GD, Mohammed AI, Paolini R, Bijlsma E, Toulson S, Holden J (2024). Chronic oral inoculation of porphyromonas gingivalis and Treponema denticola induce different brain pathologies in a mouse model of alzheimer disease. J Infect Dis.

[5] Mirmohammadsadegh N, Mashreghi Mohammadi N, Amin M (2022). Potential treponema denticola-based periodontal vaccine to resolve a global public health challenge: a narrative literature review. Expert Rev Vaccines.

[6] Satam H, Joshi K, Mangrolia U, Waghoo S, Zaidi G, Rawool S (2023). Next-generation sequencing technology: current trends and advancements. Biology (Basel).

[7] Su SS, Chen XB, Zhou LP, Lin PC, Chen JJ, Chen CS (2022). Diagnostic performance of the metagenomic next-generation sequencing in lung biopsy tissues in patients suspected of having a local pulmonary infection. BMC Pulm Med.

[8] Tiwari S, Saxena S, Kumari A, Chatterjee S, Hazra A, Choudhary AR (2020). Detection of red complex bacteria, P. gingivalis, T. denticola and T. forsythia in infected root canals and their association with clinical signs and symptoms. J Fam Med Prim Care.

[9] Holt SC, Ebersole JL (2005). Porphyromonas gingivalis, Treponema denticola, and tannerella forsythia: the “red complex”, a prototype polybacterial pathogenic consortium in periodontitis. Periodontol 2000.

[10] Li X, Kolltveit KM, Tronstad L, Olsen I (2000). Systemic diseases caused by oral infection. Clin Microbiol Rev.

[11] Rautemaa R, Lauhio A, Cullinan MP, Seymour GJ (2007). Oral infections and systemic disease--an emerging problem in medicine. Clin Microbiol Infect.

[12] Bezamat M (2022). An updated review on the link between oral infections and atherosclerotic cardiovascular disease with focus on phenomics. Front Physiol.

[13] Newman KL, Kamada N (2022). Pathogenic associations between oral and gastrointestinal diseases. Trends Mol Med.

[14] Xu B, Han YW (2022). Oral bacteria, oral health, and adverse pregnancy outcomes. Periodontol 2000.

[15] Soiniemi L, Solje E, Suominen AL, Kanninen KM, Kullaa AM (2024). The association between oral diseases and neurodegenerative disorders. J Alzheimers Dis.

[16] Ohyama H, Nakasho K, Yamanegi K, Noiri Y, Kuhara A, Kato-Kogoe N (2009). An unusual autopsy case of pyogenic liver abscess caused by periodontal bacteria. Jpn J Infect Dis.

[17] Botros N, Yan SR, Wanless IR (2014). Malakoplakia of liver: report of two cases. Pathol Res Pract.

[18] Kin LX, Butler CA, Slakeski N, Hoffmann B, Dashper SG, Reynolds EC (2020). Metabolic cooperativity between porphyromonas gingivalis and Treponema denticola. J Oral Microbiol.

[19] Foschi F, Izard J, Sasaki H, Sambri V, Prati C, Muller R (2006). Treponema denticola in disseminating endodontic infections. J Dent Res.

[20] Gu W, Deng X, Lee M, Sucu YD, Arevalo S, Stryke D (2021). Rapid pathogen detection by metagenomic next-generation sequencing of infected body fluids. Nat Med.

[21] Chen M, Lai Z, Cheng M, Liang T, Lin Z (2022). Rare brain and pulmonary abscesses caused by oral pathogens started with acute gastroenteritis diagnosed by metagenome next-generation sequencing: a case report and literature review. Front Cell Infect Microbiol.

[22] Huang J, Weng H, Ye L, Jiang M, Chen L, Li Y (2024). Bronchoalveolar lavage fluid and lung biopsy tissue metagenomic next-generation sequencing in the diagnosis of pulmonary cryptococcosis. Front Cell Infect Microbiol.

[23] Zhang Y, Chen J, Yi X, Chen Z, Yao T, Tang Z (2022). Evaluation of the metagenomic next-generation sequencing performance in pathogenic detection in patients with spinal infection. Front Cell Infect Microbiol.

